# The role of extended antral resection on weight loss and metabolic response after sleeve gastrectomy: A retrospective cohort study

**DOI:** 10.12669/pjms.36.6.2321

**Published:** 2020

**Authors:** Adem Yuksel, Murat Coskun, Kerem Karaman

**Affiliations:** 1 Adem Yuksel, Derince Teaching and Research Hospital, Department of Gastroenterological Surgery, Kocaeli, Turkey; 2 Murat Coskun, Derince Teaching and Research Hospital, Department of General Surgery, Kocaeli, Turkey; 3 Kerem Karaman Sakarya University, Faculty of Medicine, Department of Gastroenterological Surgery, Sakarya, Turkey

**Keywords:** Antral resection, Excess weight loss, Laparoscopic sleeve gastrectomy, Metabolic response, Total body weight loss

## Abstract

**Objective:**

The impact of extended antral resection (AR) after laparoscopic sleeve gastrectomy (LSG) on clinical results is still not clearly elucidated with conflicting results. Our study aimed to determine whether AR is superior to antral preservation (AP) regarding clinical results.

**Methods:**

Patients were divided into two groups according to the distance of gastric division as AR group (2cm from pylorus) and AP group (6cm from pylorus). Postoperative excess weight loss percentile (%EWL) and total body weight loss percentiles (%TBWL) at the end of first, 6^th^ and 12 months were compared. Secondly, metabolic parameters and complications were compared.

**Results:**

The first 68 patients underwent AP, and the following 43 patients underwent AR. Although statistically not significant, AR achieve more %EWL and %TBWL at the end of the first year, (P>0.05). On the other hand, metabolic parameters were similar at the end of the first year, (P>0.05). Resolution of comorbidities were statistically not different, (P>0.05). Staple line leak occurred in two patients of the AR group (4.7%) and two patients of the AP group (2.9%), (P>0.05).

**Conclusion:**

Both AR and AP seem to be equally effective in resolution of metabolic response. Although statistically not significant- AR provided more %EWL and %TBWL at the end of 12 months.

## INTRODUCTION

Laparoscopic sleeve gastrectomy (LSG) was initially designed as the first step of a two-staged bariatric procedure. However, in the following years, LSG was found as effective as other bariatric procedures and has become increasingly popular as a stand-alone bariatric procedure.^1^,^2^ Recently, LSG is the most performed bariatric procedure worldwide.^3^ The simplicity of surgical technique, preservation of the pyloric functions, none development of marginal ulcers or internal herniation, less need of trace element and vitamin supplementation are the advantages of LSG over other bariatric procedures.^4^-^6^ However, debate continues regarding the AR or AP on effective weight loss alterations and metabolic response. Further, the distance from pylorus at which the stomach division should be started is still not standardized and has been reported varying from two to 8cm.^7^-^9^

The aim of the present retrospective cohort study was to determine whether AR is superior to AP regarding weight loss and resolution of co-morbidities.

## METHODS

Following approval of the ethical committee patients who underwent LSG between January 2016 and June 2018 are retrospectively analyzed. Patients with BMI ≥ 40 were included. Patients with a history of previous bariatric surgery and patients who did not attend regular follow-up visits (first, 6^th^ and 12 months) were are excluded. (Registration: Clinical Trials.gov NCT04109664). The patients were grouped according to the distance of gastric division as AP group (6cm from pylorus) and AR group (2cm from pylorus). Sixty-eight patients underwent AP, whereas 43 patients underwent AR.

Patient characteristics and demographic data, including age, gender, BMI, co-morbid diseases (hypertension (HT), Type II diabetes, dyslipidemia), biochemical parameters (glucose, HbA1c, C-peptide, insulin, cholesterol, triglyceride, HDL, LDL, and VLDL were extracted from a prospectively prepared patient’s chart. The 30-day outcomes including postoperative morbidity and mortality are taken from patient’s folder.

Weight loss alteration at the end of first, 6^th^ and 12 months were calculated as excess weight loss percentile (%EWL) and total body weight loss percentile (%TBWL). The %EWL was calculated as [(preoperative weight – follow up weight) / (preoperative weight-ideal weight)] x100, with ideal weight based on a BMI of 25kg/m^2^. The %TBWL was calculated as [(preoperative weight – follow up weight) / (preoperative body weight)] x100.

Resolution of co-morbidities was defined as reduction of co-morbidity related symptom and signs with change of specific biochemical blood tests to normal ranges. The Clavien-Dindo classification scale was used to define the severity of complications.^10^

### Surgical Technique

The LSGs were performed by two surgeons. Patients were placed in supine position. Antibiotic prophylaxis was started before anesthesia induction. The greater omentum is carefully dissected from the stomach at a distance of 2cm for the patients with AR, and 6cm for patients with AP. All sleeves are transected using 36 French orogastric tubes. Green cartridges (4.8 mm staple height) are used for the first firing and blue cartridges (3.5 mm) for the rest. Neither over sewing sutures to the staple line nor staple line reinforcement products are used. Homeostatic metallic clips are used for bleeding at the staple line. If bleeding persists and cannot be controlled, an interrupted suture is performed at the point of bleeding. A leak test with methylene blue is performed to the gastric remnant to assess the integrity of the suture line. The procedure is completed by placing an abdominal drain just next to the staple line.

### Statistical Analysis

Data analysis was performed by using SPSS Statistics version 20.0 software (IBM Corporation, Armonk, NY, USA). Descriptive statistics for continuous variables were shown as mean ± standard deviation or median (minimum-maximum) and categorical variables were shown as the number and percentage of cases. Kolmogorov–Smirnov test is used to determine normally distributed variables. A mean ± standard deviation is used for normal distribution of continuous variables, whereas median (Interquartile Range (IQR) 25th – 75th) percentiles are used for variables without normal distribution. The significance of the difference between the groups in terms of mean was analyzed with Student’s *t* test as significance of the difference in median values was analyzed with the Mann-Whitney U test. Categorical variables were analyzed by Chi-square test, where appropriate. Finally, a *P* value *≤* 0.05 was considered statistically significant.

## RESULTS

One-hundred-forty-two patients underwent LSG. Of these, 20 patients were excluded because of different primary surgeon, four patients were excluded due to their previous bariatric surgery, and seven patients who did not attend regular follow-up visits were not included. Thus 111 patients were included to the study. The pyloric distance of antral resection was 6 cm in 68 patients (61.3%), and 2cm in the remaining 43 patients (38.7%). Ninety-two patients (82.9%) were female, and 19 patients (17.1%) were male. According to Kolmogorov-Smirnov test; there was no normal distribution in BMI, duration of the operation, length of hospital stay (hospitalization) variables. The mean age was 38.2 ± 9 years, and the median BMI was 44.5 (IQR; 41.2-49.9). No significant difference was found according to age, gender, BMI, co-morbidities (Type II diabetes mellitus, hypertension, dyslipidemia), duration of the operation and length of hospital stay, (respectively; P: 0.849, 0.942, 0.328, 0.213, 0.931, 0.335, 0.230 *P*>0.05), (Table-I).

**Table-I:** Patient’s characteristics and demographic data.

	Total (n:111)	AR (n:43)	AP (n:68)	P Value
Weight (kg)	121.7 ± 17.1	122.5 ± 19.1	121.2 ±15.9	0.695
BMI (kg/m^2^)	44.5(41.2-49.9)	44.5(41–49.9)	44.7(41.2–50)	0.861
Age (years)	38.2 ± 9	38.0 ± 9.3	38.3 ± 8.8	0.849
Gender (Male/Female)	19/92	8/35	11/57	0.942
Type II Diabetes Mellitus	44(39.6%)	20(45.5%)	24(54.5%)	0.328
Hypertension	32(28.8%)	9(28.1%)	23(71.9%)	0.213
Dyslipidemia	25(22.5%)	9(36%)	16(64%)	0.931
Operation time (min)	70(60-80)	75(60-80)	70(60-83.7)	0.335
Hospitalization (days)	5(5-6)	5(5-5)	5(5-6)	0.23

**BMI:** Body mass index

Preoperative and postoperative first year fasting glucose, HbA1c, insulin, C-peptide, cholesterol, triglyceride, HDL, LDL, VLDL levels were not statistically different in two groups, (*P*>0.05), (Table-II).

**Table-II:** Comparison of metabolic parameters.

	AR (n:43)	AP (n:68)	P Value
***Before Surgery***			
Glucose	124.6 ± 49.6	121.2 ± 15.9	0.082
HbA1c	6.3 ± 1.4	6.1 ± 0.8	0.976
Insulin	53.8 ± 13.8	56.7 ± 9.6	0.357
C-peptide	2.7 ± 1.5	3 ± 1.2	0.035
Cholesterol	196.9 ± 33.3	192.6 ± 34.8	0.521
Triglyceride	154 ± 85	158.5 ± 72.9	0.769
HDL	49.3 ± 9.9	47 ± 8.5	0.198
LDL	116 ± 25.8	113.6 ± 29.3	0.658
VLDL	28.8 ± 11.7	31.2 ± 14.9	0.575
***12 Months***			
Glucose	87.7±9.5	89.3±8	0.624
HbA1c	5.2±0.3	5.2±0.4	0.402
Insulin	6.5±4.3	8.7±8.6	0.068
C-peptide	1.2±0.5	1.3±0.5	0.551
Cholesterol	193±28.2	189.4±34.2	0.515
Triglyceride	99.6±38.7	87.4±35.9	0.115
HDL	53.8±13.8	56.8±9.7	0.228
LDL	118.8±24.8	115.2±29.4	0.508
VLDL	19.9 ± 7.7	17.3±7.23	0.076

**HbA1c:** Glycated hemoglobin A1c, **HDL:** High-density lipoprotein,

**LDL**: Low-density lipoprotein, **VLDL:** Very-low-density-lipoprotein.

Postoperative mean %EWL at the end of first, 6^th^, and 12 months of the AP group were in order 22.7±8.1%, 57.1±2% and 69.6±20.5%, respectively. On the other hand, postoperative mean %EWL at the end of first, 6^th^, and 12 months of the AR group were 24.2±8.5%, 59±16.9% and 72.6±20.6%, respectively. When compared the two group regarding %EWL, no significant difference has been found, (*P*>0.05), (Table-III).

**Table-III:** Comparison of the groups according to EWL% and TBWL%.

	Total (n: 111)	AR (n: 43)	AP (n: 68)	P values
**%EWL**				
at 1 months	23.3 ± 8.2	24.2 ± 8.5	22.7 ± 8.1	0.654
at 6 months	57.8 ± 17.5	59 ± 16.9	57.1 ± 2	0.584
at 12 months	70.8 ± 20.5	72.6 ± 20.6	69.6 ± 20.5	0.456
**%TBWL**				
at 1 months	10.1 ±2.8	10.5 ± 2.8	9.9 ± 2.8	0.272
at 6 months	25.3 ± 5.8	25.9 ± 5.8	24.9 ± 5.8	0.368
at 12 months	31.3 ± 7.6	32.1 ± 8	30.8 ± 7.4	0.372

%**EWL:** Excess weight loss percentile, %**TBWL:** Total body weight loss percentile.

Postoperative mean %TBWL at the end of first, 6^th^, and 12 months of the AP group were in order 9.9±2.8%, 24.9±5.8%, and 30.8±7.4 respectively. On the other hand, postoperative mean %TBWL at the end of first, 6^th^, and 12 months of the AR group were 10.5±2.8%, 25.9±5.8% and 32.1±8%, respectively. The difference between the two groups according to %TBWL was not significant, (respectively; p: 0,272, 0,368, 0,372 *P*>0.05), (Table-III).

Thirty-day morbidity rate was not statistically different in two groups (*P*>0.05). Staple line leak occurred in 2 patients (2.9%) of the AP groups, and 2 patients (4.6%) of the AR group, respectively. Of the AP group, gastric leak healed spontaneously in one patient after 10 days and in the remaining patient 15 days later under conservative management with total parenteral nutrition and percutaneous drainage. On the other hand, leaks of the AR group healed in order 3 and 6 months later with gastric stent placement. Mortality did not occur. None of the patients had major complains of gastroesophageal reflux disease (GERD) requiring hospitalization or surgical intervention. Although statistically not significant, the complication rate was higher in the AR group (18.6%) than the AP group (11.8%), (*P*>0.05), (Table-IV).

**Table-IV:** Postoperative complications.

	AR (n:43)	AP (n:68)
***Clavien-Dindo Grade***		
I	2	1
II	4	7
III	2	0
IV	0	0
V	0	0

Total; n (%)	8 (18.6)	8 (11.8)

When compared the two groups according the resolution of HT, Type-II DM, and dyslipidemia no significant difference was found at the end of the first year, (*P*>0.05), (Table-V).

**Table-V:** Resolution of co-morbidities at the end of first year.

	AR (n: 43)	AP (n: 68)	P values
Type 2 Diabetes, n (%)	17/20 (85%)	22/28 (78.6%)	0.716
Hypertension, n (%)	9/10 (90%)	17/22 (77.3%)	0.637
Dyslipidemia, n (%)	5/9 (55.6%)	10/16 (62.5%)	1.0

## DISCUSSION

The main approach to include antrum within resection margins during LSG is to maintain further reduction of gastric volume with the hope of more effective long lasting weight loss. However, some authors claim that preserving the antrum is crucial for contractile function that promotes gastric emptying. Further, a preserved antrum may decrease the risk of leakage by reducing intragastric pressure and eventually, gastro-esophageal reflux.^11^-^13^

Results of several studies regarding the effect of AR on weight loss is controversial.^7^,^8^,^14^-^16^ Abdallah et al.^7^, observed in their study that extended antral resection (division at 2cm from the pylorus) provides significantly more %EWL at 6, 12 and 24 months than the division at 6cm. On the other hand, Garay and his colleagues,^8^ found no significant difference after one-year follow-up regarding %EWL between AR (division at 2cm from the pylorus) and AP (division at 5cm from the pylorus). Further, they observed significantly accelerated gastric emptying in antrum preserved patients when compared with extended antral resection performed ones. ELGeidie et al.^14^, found no significant %EWL at 12 months between AR (division at 2cm from the pylorus) and AP (division at 6cm from the pylorus) groups. Obeidat and his colleagues,^15^ achieved significantly more %EWL in extended antrum resected patients (transection 2cm from pylorus) at the first and second years than the AP group (transection 6cm from the pylorus). Further, although not significant- weight regain occurred more frequent in the AP group. Yormaz et al.^16^, found significantly more %EWL and %TBWL in the AR group (division at 2cm from the pylorus) at 6, 12 and 24 months when compared with the AP group (division at 6cm from the pylorus). Avlanmis et al.^17^, observed that a resection margin with a short distance (<3cm) to pylorus is associated with better %EWL during 36 months. However, they also observed that these patients are more prone to nausea and vomiting in the early postoperative period. In the present study, following the first 68 consecutive patients with AR, we began to AR with the hope of achieving more long lasting weight loss. Although statistically not significant- AR provided more %EWL and %TBWL at the end of 12 months. The statistically insignificance may probably relate to the small sample size.

It has been previously shown that bariatric surgery effectively achieves remission of type- II DM and obesity related comorbidities.^18^ The effect of LSG as a bariatric procedure has also been proven in achieving effective weight loss.^19^ On the other hand, resection of antrum on metabolic response is also still not clearly elucidated with often similar results.^7^,^13^,^14^,^20^ Abdallah et al.^7^, observed no significant difference between AR and AP groups in resolution of co-morbidities. Khalifa et al.^13^, found no significant difference between AR (division at 2cm from the pylorus) and AP (division at 6cm from the pylorus) groups in resolution of Type-II diabetes and HT at 6 months. ELGeidie and his collagenous^14^ compared between AR and AP groups the resolution of Type-2 diabetes, HT and dyslipidemia at 6 and 12 months. They found similar results. Vives and his collagenous^20^ compared metabolic parameters at 12 months between AR (division at 3cm from the pylorus) and AP (division at 8cm from the pylorus) groups including c-peptide, insulin, HbA1c, glucagon like peptide-1 (GLP-1) and gastric inhibitory peptide (GIP). They found no difference after one year according to GLP and GIP changes. Hyperinsulinemia significantly improved in the AR with respect to AP group but only in diabetic patients. In the present study, both parameters of hyperinsulinemia (glucose, insulin, HbA1c, and C-peptide) and dyslipidemia (cholesterol, triglyceride, HDL, LDL, and VLDL) were similar between AR and AP groups at the end of 12 months.

An effective approach to LSG aims to eliminate complications while achieving maximum weight loss with resolution of comorbidities. Theoretically, the risk of staple line leak increases as the remained gastric volume decreases. Notwithstanding, several studies showed that the risk doesn’t increase with AR.^7^,^8^,^14^,^21^ In the present study, staple line leak occurred in two patients from the AP group (2.9%) and two patients from the AR group (4.7%), respectively. Similar to the literature data, the complication rates of the present study were statistically equal both in AR and AP performed patients. However, the healing process of the leaks took longer time with AR when compared to AP. This is most likely related to a much narrower gastric lumen with higher intragastric pressure after AR which makes difficult to close and heal of the leak defect.

### Limitations of the study

First, it is retrospective. Secondly, the sample size is small. Third, long term results are still not known. On the other hand, besides the weight loss alterations, the study provides also detailed information on changes of metabolic parameters at the end of the first postoperative year.

## CONCLUSION

LSG appears to be an effective bariatric procedure in improving metabolic parameters as well as weight loss. Both LSG with AR and AP are equally effective in resolution of metabolic response at the end of the first year. On the other hand, AR seems to achieve more %EWL and %TBWL than the AP.

### Authors’ Contributions:

**AY:** Concept, study design, data collection and processing, data interpretation, statistical analysis, literature search, writing and is responsible for integrity of research.

**MC:** Data collection, literature search.

**KK:** Concept, data interpretation, critical review.

## References

[1] Shi X, Karmali S, Sharma AM, Birch DW (2010). A review of laparoscopic sleeve gastrectomy for morbid obesity. Obes Surg.

[2] Peterli R, Wölnerhanssen BK, Vetter D, Nett P, Gass M, Borbély Y (2017). Laparoscopic sleeve gastrectomy versus Roux-Y-gastric bypass for morbid obesity—3-year outcomes of the prospective randomized Swiss Multicenter Bypass Or Sleeve Study (SM-BOSS). Ann Surg.

[3] Angrisani L, Santonicola A, Iovino P, Vitiello A, Higa K, Himpens J (2018). IFSO Worldwide Survey 2016:Primary, endoluminal, and revisional procedures. Obes Surg.

[4] Zellmer JD, Mathiason MA, Kallies KJ, Kothari SN (2014). Is laparoscopic sleeve gastrectomy a lower risk bariatric procedure compared with laparoscopic Roux-en-Y gastric bypass?A meta-analysis. Am J Surg.

[5] Zhang Y, Ju W, Sun X, Cao Z, Xinsheng X, Daquan L (2015). Laparoscopic sleeve gastrectomy versus laparoscopic Roux-en-Y gastric bypass for morbid obesity and related comorbidities:a meta-analysis of 21 studies. Obes Surg.

[6] Guerrier JB, Dietch ZC, Schirmer BD, Hallowell PT (2018). Laparoscopic sleeve gastrectomy is associated with lower 30-day morbidity versus laparoscopic gastric bypass:an analysis of the American College of Surgeons NSQIP. Obes Surg.

[7] Abdallah E, El Nakeeb A, Yousef T, Abdallah H, Ellatif MA, Lotfy A (2014). Impact of extent of antral resection on surgical outcomes of sleeve gastrectomy for morbid obesity (a prospective randomized study). Obes Surg.

[8] Garay M, Balagué C, Rodríguez-Otero C, Gonzalo B, Domenech A, Pernas JC (2018). Influence of antrum size on gastric emptying and weight-loss outcomes after laparoscopic sleeve gastrectomy (preliminary analysis of a randomized trial). Surg Endosc.

[9] Pereferrer FS, López AM, Espelta MV, Carceller ER, Blasco SB, Vilalta FB (2017). Weight loss analysis according to different formulas after sleeve gastrectomy with or without antral preservation:A randomised study. Obes Surg.

[10] Dindo D, Demartines N, Clavien P-A (2004). Classification of surgical complications:a new proposal with evaluation in a cohort of 6336 patients and results of a survey. Ann Surg.

[11] Nakane Y, Michiura T, Inoue K, Sato M, Nakai K, Yamamichi K (2002). Length of the antral segment in pylorus-preserving gastrectomy. Br J Surg.

[12] Robert M, Pasquer A, Pelascini E, Valette P-J, Gouillat C, Disse E (2016). Impact of sleeve gastrectomy volumes on weight loss results:a prospective study. Surg Obes Relat Dis.

[13] Khalifa IG, Tobar WL, Hegazy TO, Balamoun HA, Mikhail S, Salman MA (2019). Food tolerance after laparoscopic sleeve gastrectomy with total antral resection. Obes Surg.

[14] ElGeidie A, ElHemaly M, Hamdy E, El Sorogy M, AbdelGawad M, GadElHak N (2015). The effect of residual gastric antrum size on the outcome of laparoscopic sleeve gastrectomy:A prospective randomized trial. Surg Obes Relat Dis.

[15] Obeidat F, Shanti H, Mismar A, Albsoul N, Al-Qudah M (2015). The magnitude of antral resection in laparoscopic sleeve gastrectomy and its relationship to excess weight loss. Obesity surgery.

[16] Yormaz S, Yılmaz H, Ece I, Yılmaz F, Sahin M (2017). Midterm clinical outcomes of antrum resection margin at laparoscopic sleeve gastrectomy for morbid obesity. Obes Surg.

[17] Avlanmis O, Isil RG, Burcu B (2019). Effect of Resection Distance from Pylorus on Weight Loss Outcomes in Laparoscopic Sleeve Gastrectomy. Obes Surg.

[18] Wazir N, Arshad MF, Finney J, Kirk K, Dewan S (2019). Two Years Remission of Type 2 Diabetes Mellitus after Bariatric Surgery. J Coll Physicians Surg Pak.

[19] Siddiq G, Aziz W, Pervez MB, Haider MI, Hussain SV, Khan N (2016). Early Laparoscopic Sleeve Gastrectomy Outcomes in Terms of Weight Loss. J Coll Physicians Surg Pak.

[20] Vives M, Molina A, Danús M, Rebenaque E, Blanco S, París M (2017). Analysis of gastric physiology after laparoscopic sleeve gastrectomy (LSG) with or without antral preservation in relation to metabolic response:A randomised study. Obes Surg.

[21] El Chaar M, Stoltzfus J (2018). Assessment of sleeve gastrectomy surgical technique:first look at 30-day outcomes based on the MBSAQIP database. J Am Coll Surg.

